# Fused Projection-Based Point Cloud Segmentation

**DOI:** 10.3390/s22031139

**Published:** 2022-02-02

**Authors:** Maximilian Kellner, Bastian Stahl, Alexander Reiterer

**Affiliations:** 1 Fraunhofer Institute for Physical Measurement Techniques IPM, 79110 Freiburg, Germany; bastian.stahl@ipm.fraunhofer.de (B.S.); alexander.reiterer@ipm.fraunhofer.de (A.R.); 2 Department of Suistainable Systems Engnineering INATECH, Albert Ludwigs University Freiburg, 79110 Freiburg, Germany

**Keywords:** point cloud segmentation, supervised learning, point cloud projection

## Abstract

Semantic segmentation is used to enable a computer to understand its surrounding environment. In image processing, images are partitioned into segments for this purpose. State-of-the-art methods make use of Convolutional Neural Networks to segment a 2D image. Compared to that, 3D approaches suffer from computational cost and are not applicable without any further steps. In this work, we focus on semantic segmentation based on 3D point clouds. We use the idea to project the 3D data into a 2D image to accelerate the segmentation process. Afterward, the processed image gets re-projected to receive the desired result. We investigate different projection views and compare them to clarify their strengths and weaknesses. To compensate for projection errors and the loss of geometrical information, we evolve the approach and show how to fuse different views. We have decided to fuse the bird’s-eye and the spherical projection as each of them achieves reasonable results, and the two perspectives complement each other best. For training and evaluation, we use the real-world datasets SemanticKITTI. Further, we use the ParisLille and synthetic data generated by the simulation framework Carla to analyze the approaches in more detail and clarify their strengths and weaknesses. Although these methods achieve reasonable and competitive results, they lack flexibility. They depend on the sensor used and the setup in which the sensor is used.

## 1. Introduction

With an increasing number of 3D sensors available, such as Light Detection and Ranging (LiDAR), the demand for 3D data processing is also increasing. The range of interest extends from autonomous driving [1,2,3] to infrastructure mapping [4,5,6,7] up to biomedical analysis [8].

The task of understanding a scene is challenging for a computer. It can be divided into classification, object recognition, semantic, and instance segmentation. The research community has made extreme progress in recent years for 2D semantic segmentation on images [9,10,11,12,13,14,15,16,17,18]. Methods for 3D processing on point clouds, on the other hand, are rarer and suffer from computational cost and inaccuracy. The progress achieved in the field of image processing cannot be applied without further ado. There are a few main differences between images and point clouds. First of all, point clouds are very sparse compared to images. Furthermore, the density of points within the same point cloud may vary. Commonly, the point density close to the sensor is denser than the point density further away from it. Thirdly, point clouds are irregular. This means the number of points within a respective point cloud differs. Moreover, point clouds are unstructured, which implies that each point is independent, and the distance between adjacent points varies. In addition to that, point clouds are unordered and invariant to permutation.

To circumvent the problems described above, we work with projection-based methods. This paper can be divided into three parts. First, we show different projection-based approaches and how they can be used. Instead of working with a single view, we examine the spherical, the bird’s eye, and the cylindrical views. Stating their advantages and disadvantages allows us to compare the views with each other and shows how to improve them. By using multiple projection planes as feature maps and combining them with dimensionality reducing filters, the bird’s-eye and the cylindrical view can be drastically improved. The second part is about fusing multiple views. Complementary views allow the avoidance of errors that occur, such as occlusion. This means we can improve the overall performance. To investigate the fusion process itself, we define our baseline model and compare it to non-learnable and learnable approaches. Within the third part, we answer the question about generalization. This does not only mean to apply the approaches to unseen data but also to a new sensor with a different setup, as well as to synthetic generated data.

This paper makes three major contributions:

- comparing different projection-based methods with each other to highlight the advantages and disadvantages
- improving the performance for the regular bird’s eye and cylindrical view
- proposing methods that can be used to fuse multiple projections with each other to improve the overall performance

## 2. Related Work

There are a lot of different approaches to tackle the task of 3D segmentation today. A general overview is given in [19]. Mainly, these approaches can be divided into projection-based, discretization-based, and point-based methods.

The idea of the projection-based method is to map the 3D data into a 2D image. The point cloud is transformed into a spherical image in [20,21] and SalsaNext [22]. Each of them uses a different 2D backbone for the segmentation, and both use k Nearest Neighbors (kNN) for post-processing the re-projected prediction. Instead of a spherical image, a bird’s-eye view image is used in [23]. In addition to that, Polar coordinates are used instead of Cartesian coordinates and an MLP to learn a fixed-size representation. There are already several works that fuse multiple projections. The authors of [24] extract features from both views and use an early fusion approach. Segmenting the spherical image first and projecting the results into the bird’s-eye view for further processing is done in [25]. Ref. [26] separates the two projections and adds up the resulting probabilities. In [27], the late fusion is learned by an MLP. Multiple different projections are used in [28] to perform shape segmentation. All of the named approaches have achieved excellent results. The main advantage of this method is that well-studied 2D convolution can be applied. This makes the approach very fast and accurate at the same time. Nevertheless, this approach suffers from discretization errors and occlusion. Furthermore, it highly depends on the used sensor.

The discretization-based method converts the 3D point cloud into a discrete representation. This could be voxels, octrees, or lattices, for example. Transforming the point cloud into a set of occupancy voxels is done in [29]. This way, they avoid suffering from sparsity. All points within one voxel get assigned the same label as the voxel itself after voxel-wise segmentation. Ref. [30] adopts sparse tensors and generalizes sparse convolution to work faster on sparse volumetric data. Preventing sparsity by storing feature representations within the leaves of octrees is done in [31]. Ref. [32] embeds the point cloud into sparse lattices. The convolution is applied to the lattices, and the learned features are projected back to the point cloud. First, employing cylindrical partition and then applying sparse, asymmetrical 3D convolution is performed in [33]. Even though all of these approaches achieve good results in terms of accuracy, the main problem is the computational complexity.

Point-based methods directly work on point clouds. Due to the fact that they are unstructured and orderless, regular convolution can not be applied. These methods make use of pointwise Multilayer perceptron (MLP), generalized point convolution, or graph structures. Ref. [34] was the first architecture using MLP’s to learn 3D features. In RandLA-Net [35], the same method is used to downsample the point cloud aggressively. This way, the approach can be applied to larger point clouds. Others adapt the convolution in some way to make it applicable to point clouds, such as [36], KPConv [37], or [38]. Ref. [39] represents the point cloud as a graph. By doing so, graph convolution gets applied. These approaches have achieved outstanding results in some cases as well. Unfortunately, they are limited to a maximum number of points due to their memory consumption, and they usually cannot be applied in real-time.

Moreover, there is an increasing amount of methods that fuse multiple of the above-mentioned approaches. To allow for more efficient large-scene processing, voxel-based and point-based learning get combined in [40] and in [41]. All three approaches get combined in [42]. These methods currently are leading the benchmark [43].

**Figure 1:**
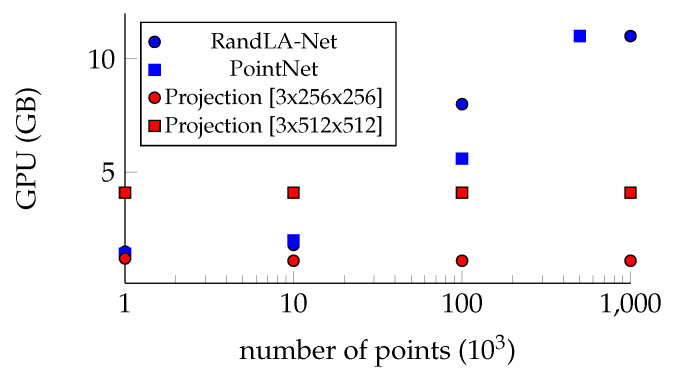
Memory consumption over number of points for point-based and projection-based methods.

All of the above approaches have their advantages and disadvantages. However, if compared in terms of runtime and memory consumption, the projection-based approaches have a clear advantage. Figure 1 shows how the memory increases for point-based methods. In comparison, the memory requirement for the projection-based methods remains the same since it does not depend on the number of points but on the projection size. In our example, we used an image of size [3 × 256 × 256] and [3 × 512 × 512] jointly with a U-Net [11] architecture. By working with point clouds with more than 100 k points, the projection-based methods need, by far, less memory.

In summary, it can be said that the projection-based approaches are the fastest. However, the geometric shapes are ignored, and discretization errors and occlusion of points occur. By using a discrete 3D representation, the geometric information can be retained, but the computational cost increases. For example, even if sparse tensors are used, the memory for the learned filters still increases in a cubic way instead of quadratic. Furthermore, discretization errors also occur with these approaches. Point-based methods avoid these errors by working without discretization steps, which is one of the biggest advantages. Currently, however, the number of points to be processed is a limiting factor. The fusion of complementary methods is a good way to balance the weaknesses of one method with the strengths of another. As the complexity increases, the methods should be chosen carefully to achieve the best ratio for the complexity and performance.

## 3. Projection-Based Methods

We focus on three different views that are going to be described shortly. All of them share the idea of mapping the point cloud $R^{3}\mapsto R^{2}$. Projecting the 3D point cloud into a 2D image brings some significant advantages. First, the resulting image is always the same size, making it easier to train a model and combine the images into batches. Secondly, the projection is structured again. Thus, we know the neighboring pixels, and the well-studied 2D convolution can be applied, allowing for feature learning. This makes the model fast and keeps the required memory lower than 3D convolutions, as the number of parameters for the kernel grows only in a quadratic way instead of cubic. There are some disadvantages on the other hand. Due to the projection, we lose 3D geometric information. By ordering the point cloud and projecting them into an image, discretization errors occur, and points can overlap. This leads to occlusion errors and aggravates projecting the segmented points back to the point cloud.

### 3.1. Spherical View

To use this approach, the Cartesian coordinates get initially mapped into spherical coordinates. Afterward, the points get discretized into a spherical image $I\in R^{h\times w}$, with *w* and *h* representing the width and height of the projected image. The mapping is described in the following:

(1) $$\left(\begin{array}{c} u\\ v \end{array}\right)=\left(\begin{array}{c} \frac{1}{2}\left(1-\frac{\phi }{\pi }\right)w\\ \left(1-\frac{\theta +f_{down}}{f}\right)h \end{array}\right)$$

The vertical field of view $f=|f_{down}\left|+\right|f_{up}|$ depends on the sensor. The values for *u* and *v* are holding the image coordinates for each point. The resulting tensor has the shape of $\left[c_{in}\times h\times w\right]$ with a $c_{in}$ feature channel. The input features might be the depth *d*, if given the intensity *i*, the coordinates *x*, *y*, and *z*, or surface normals *n*. Since multiple points can be within one coordinate tuple, the points are ordered descending. This means that only the features for the closest point within a tuple are used.

By knowing the point coordinates and considering the image holding them as a function z=f(x,y), we can calculate a normal from the parametrization r(x,y)=(x,y,z)=(x,y,f(x,y)), given:

(2) $$\begin{array}{cc} n & =\frac{\partial r}{\partial x}\times \frac{\partial r}{\partial y}\\ \; & =\left(1,0,\frac{\partial f}{\partial x}\right)\times \left(0,1,\frac{\partial f}{\partial y}\right)\\ \; & =\left(-\frac{\partial f}{\partial y},-\frac{\partial f}{\partial x},1\right) \end{array}$$

The last equation can be implemented efficiently by using a GPU and a high abstraction deep learning framework.

As this type of projection is the most developed one, we will use the architecture from [22]. We focus on investigating the input features and the size of the image itself. For the Velodyne HDL-64E, the angular resolution is 0.08° and the vertical approximately 0.4°. The horizontal field of view is 360°, and the vertical is 26.9°. This means by using an image width $w=\frac{360}{0.08}=4500$ and an image height $h=\frac{26.9}{0.4}$∼67, we are theoretically able to project every point into the image without problems, such as occluding points. Nevertheless, the computational effort increases and the image becomes more sparse. For this reason, we are interested in keeping the image small.

### 3.2. Bird’s-Eye View

The idea of this approach is to collapse the point cloud and project it into the ground plane, which most commonly is the x−y plane. To find the plane to project on, we are using the RANdom SAmple Consensus (RANSAC) algorithm [44], and we use it to normalize the points. We discretize the point cloud into multiple planes instead of a single one to avoid suffering from too much occlusion. The resulting tensor has the shape of $\left[c_{in}\times h\times w\right]$ with $c_{in}$ height channels.

**Figure 2:**
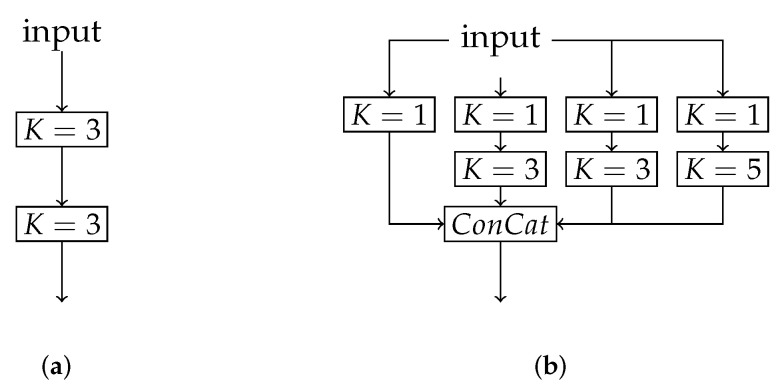
Double convolution and adapted inception module for U-Net. One filter branch with a Kernel of K=3 is dilated by D=2. Batch normalization and ReLu as the activation function is used after each convolution. (**a**) Regular U-Net; (**b**) Adapted inception module.

We use U-Net [11] as our base network architecture. To combine the feature maps with each other and to increase the receptive filter field, we replace all double convolution blocks, but the input and output one, with an adapted version of the inception module [45]. Instead of the max-pooling branch, we use another convolution one with a kernel of K=3, which is dilated by D=2. The padding size of each branch is chosen to allow for equivalent output sizes. Each block is followed by a batch normalization layer and ReLu as an activation function. All blocks with K=1 that are followed by another convolution reduce the amount of feature channels by a factor of 8 compared to the desired overall output channels. The convolution block afterward doubles the feature map again. The solo reducing filter quarters the feature channels. Concatenating all branches ends up in the desired output size. This approach reduces the trainable parameters by a factor of approximately 10 compared to the regular U-Net. In Figure 2 both modules are visualized.

### 3.3. Cyclindrical View

Mapping some given point cloud into a cylindrical image is similar to Equation (1). As most used LiDAR sensors are spherical by nature, this method is not commonly used. Nevertheless, we have investigated this view for the purpose of completeness and to avoid the disadvantage of deforming physical dimensions caused by the spherical view. Since occlusion is highly relevant for this approach, we use the same idea and architecture as for the bird’s-eye approach. Instead of using $c_{in}$ height maps, we divide the radial distance ρ and use it as our feature input.

## 4. Fusion

**Figure 3:**
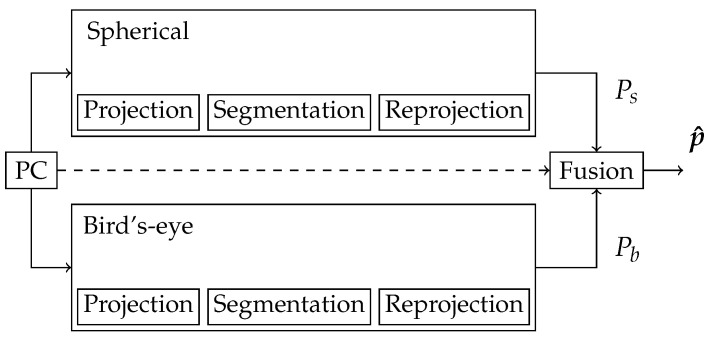
Basic architecture of combining different approaches. Each branch handles the whole process on its own. Only the prediction matrices for all points $P_{s}$ and $P_{b}$ are used within the fusion. Optionally, the coordinates of the points themselves can also be included in the fusion.

Occlusion is considered to be one of the main problems with projections. This means objects captured by the sensor behind some other object can be covered within the projection. During re-projection, the covered object will get assigned the same label as the object in the foreground. Most of the work published so far uses Nearest Neighbor (NN) methods to deal with this error. We decrease this error by combining different projections. This is also one reason we chose this work to deal only with the late fusion approach. The process is illustrated in Figure 3. The following describes how the fusion block can look like.

### 4.1. Baseline

**Figure 4:**
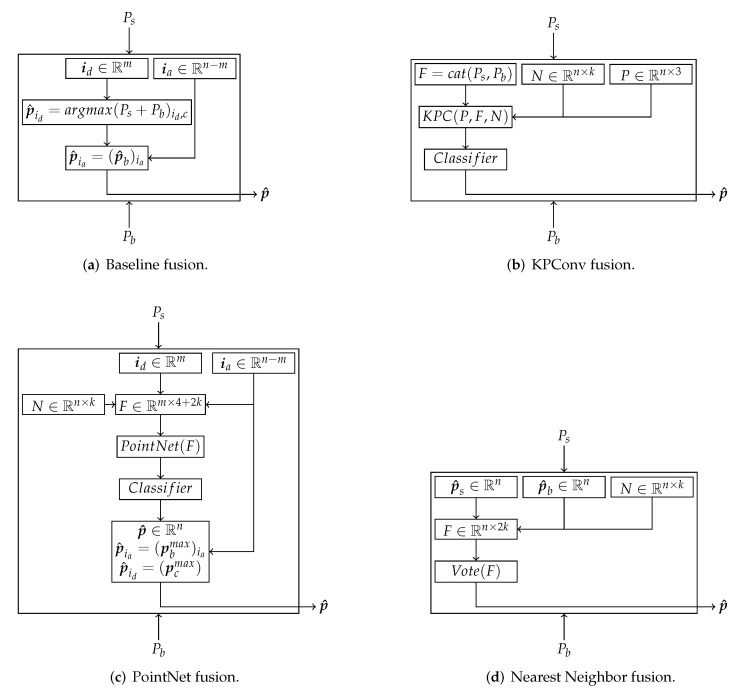
Different fusion approaches illustrated. Within the baseline (**a**) predictions, where both branches agree, are accepted directly. The probabilities for belonging to a class are added, and the class with the highest value is assigned in case of mismatched predictions. KPConv fusion (**b**) uses the NN, the point coordinates, as well as the predictions as features to learn the fusion. PointNet fusion (**c**) works similar, but works only on disagreeing points, and for the fusion process, a small PointNet network is used. Within the NN fusion (**d**), a consensus vote is performed over the NN and both predictions. All but (**a**) make use of the point coordinates.

As baseline, we use an approach similar to [26], but as we try to avoid errors by using different perspectives, we do not use any individual post-processing. To allow for easier investigation, the outputs for each branch get divided into agreeing and disagreeing predictions. The indices for agreeing points are defined as $\left\{i_{a}\in R^{n-m}:{\hat{p}}_{s}={\hat{p}}_{b}\right\}$ and for disagreeing $\left\{i_{d}\in R^{m}:{\hat{p}}_{s}\neq {\hat{p}}_{b}\right\}$. Both branches can predict reasonable results on their own. This means that in cases in which both branches predict the same label for one point, this prediction is most likely correct. This might come in handy for later calculations as it can be used to reduce the amount of points. Our baseline fusion can be seen in Figure 4a. The matrices $P_{s}$ and $P_{b}$ hold the whole output of each branch and $\hat{p}$ the final predicted class.

### 4.2. KPConv Fusion

The KPconv [37] is a convolution specially designed for point clouds. The layer takes the points $P\in R^{n\times 3}$, the corresponding features $F\in R^{n\times d_{in}}$, and the neighborhood indices $N\in R^{n\times k}$ as inputs. This convolution brings further hyperparameters into the model. For the first experiments, the number of kernel points, the radius used for the kernel points, and the radius’s influence are set to values recommended by the paper.

In our case, we have used $d_{in}=40$ as the feature dimension, using the whole prediction outputs $P_{s}$ and $P_{b}$. A KD-Tree, implemented within the scikit−learn library [46], is used for searching the NN. The hyperparameter for the amount of neighbors is set to k=8. A 1×1 convolution-based classifier map for each feature vector to the number of classes. The fusion scheme is visualized in Figure 4b.

### 4.3. PointNet Fusion

PointNet [34], which is based on MLP structures, can be used to fuse these predictions. Only a simplified version has been applied to avoid blowing up the fusion. At first, the predictions get split into agreeing and disagreeing, as described earlier. A KD-Tree is used to calculate the *k* neighbors of the point cloud, and a new feature tensor $F\in R^{m\times 4+2k}$ gets created. It only contains the points, intensity values in which both branches disagree, and the predictions of both branches for the *k* neighborhood. This allows only to recalculate the predictions where the branches are uncertain and keep the computational cost low. This approach is visualized in Figure 4c.

### 4.4. Nearest Neighbor Fusion

For this approach, we fuse the projections based on the nearest neighbors (NN). The NN method has proven to achieve reasonable post-processing results. However, instead of avoiding projection errors, we use them to make a consensus vote for taking the label that appears the most often within the defined region. This is done by creating a new feature matrix $F\in R^{n\times 2k}$ that contains the predictions from both branches, as well as the predictions for the *k* NNs. The procedure is shown in Figure 4d.

## 5. Experiments

### 5.1. Evaluation Metric

For the evaluation of each model, we use the mean intersection-over-union (mIoU) given by:

(3) $$mIoU=\frac{1}{C}\sum_{c\in C}\frac{|P_{c}\cap G_{c}|}{|P_{c}\cup G_{c}|}$$

with the class prediction $P_{c}$ and the class ground truth $G_{c}$.

### 5.2. Datasets

We use three different datasets for training and evaluation. The SemanticKITTI [43] and the ParisLille [47] as real-world datasets and the simulation framework Carla [48] to generate synthetic data. SemanticKITTI is used for training, and the other ones are used for generalization and knowledge transfer causes.

SemanticKITTI is a large dataset that uses a Velodyne HDL64 LiDAR mounted on top of a car pointing forward. The data were collected by KITTI [49] in the metropolitan area of Karlsruhe. It contains more than 43 k scans that are divided into 22 sequences. The first half is commonly used for training and the second half for testing. Sequence number 8 is used for validation purposes. First, all frames are pointwise labeled into 28 classes. Next, the moving classes are merged with the non-moving classes, which leaves 19 classes, ignoring the unlabeled points.

Carla is an open-source simulation framework that has been developed for autonomous driving purposes. Since the setup and adaption of sensors are highly flexible, Carla can quickly generate training data. There are eight different maps available. Each map offers a different environment. For collecting data, the simulated sensor gets mounted on top of the car. The sensor can be adapted to comply with the specification given by the actual sensor. We created the same sensor as used in the SemanticKITTI dataset. While the car drives automatically through the environment, every time the car has traveled a distance longer than some threshold, a point cloud gets saved. This procedure is used to guarantee diverse point clouds.

ParisLille is, again, a real-world dataset. A Velodyne HDL32 Lidar, mounted at the back of the car, facing downwards, is used. Multiple scans are mapped together, which results in three large sequences (Lille1, Lille2, and Paris). All points are labeled into 50 different classes. For easier usage, we split each sequence back into the original scans and transform the annotation. The raw data is used to gather the necessary information. These provide the spherical angles θ and ϕ, as well as the origins of the sensor during recording. The raw data is not annotated. Therefore, we have to search for the nearest neighbor within the training data to assign the right labels. To make future comparisons easier, we further map the labels into the SemanticKITTI definition. The described procedure ends with eight sequences for Lille1, three for Lille2, and five for Paris.

We chose these three datasets for the following reasons. First, SemanticKITTI is used since it contains the most labeled points. Training will be performed on this dataset. ParisLille is selected since the sensor setup and the sensor itself differ. This allows evaluating if it is possible to transfer the learning process and the generalization for the methods. Lastly, Carla offers great flexibility with regard to collecting data in any setup without the need for labeling.

### 5.3. Training Details

All models and the whole training pipeline is implemented using pytorch [50]. As the classes within the datasets are highly imbalanced, we will use a weight for each class $w_{c}=\frac{1}{\sqrt{f_{c}}}$ leading to the weighted cross-entropy loss $L_{\mathrm{wce}}$:

(4) $$L_{\mathrm{wce}}=-\frac{1}{\sum_{c\in C}w_{c}}\sum_{c\in C}w_{c}y_{c}\log{\hat{y}}_{c}$$

The Lovász-Softmax loss $L_{\mathrm{ls}}$ [51] allows for optimizing the IoU metric. It is defined as:

(5) $$L_{\mathrm{ls}}=-\frac{1}{\left|C\right|}\sum_{c\in C}\Delta_{J_{c}}\left(e\left(c\right)\right)$$

where e(c) is the vector of pixel errors for class *c* and $\Delta_{J_{c}}$ is the Lovász extension of the IoU.

To optimize for the pixel wise accuracy and the IoU, we use a linear combination of both losses $L=L_{\mathrm{wce}}+L_{\mathrm{ls}}$.

Stochastic gradient descent (SGD) [52] is used as an optimizer. We followed [53] to estimated reasonable boundary values for the one cycle learning rate [54] and used a momentum β within the range (0.95,0.85). The L2 penalty is set to 0.0001, and a dropout probability of 0.2 is applied.

To avoid overfitting, the data gets augmented. First, we drop a random amount of U(0,0.3) points. Afterward, the *x* and *y* position of each point gets shifted by the value of U(−5,5), and the point cloud gets rotated around the *z*-axis by an angle between 30° and 330°. For the bird’s-eye view, the *z* position of each point gets shifted by U(−1,1) additionally. Each augmentation but the first gets applied independently with a probability of 0.5. Note that all augmentation is applied to the point cloud and not the projection.

### 5.4. Single Projection

#### 5.4.1. Spherical View

Table 1 clearly shows that it is not necessary to project the points themselves into the image. Even by only using the depth, the performance is quite good. The best results were achieved by the network, which uses the normals as an additional feature. It has to be taken into account that this model takes slightly more time for the projection itself.

**Figure 5:**
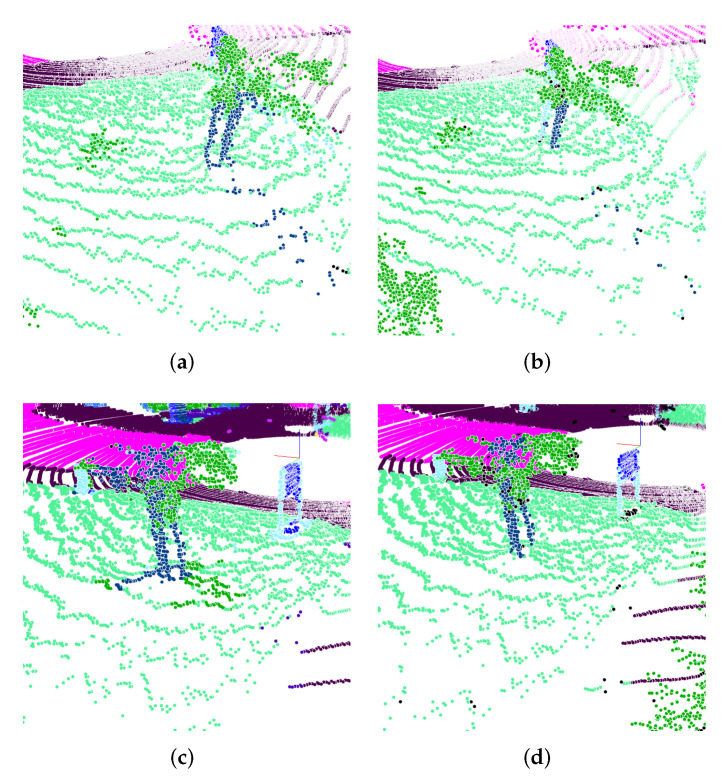
Projection error for different cases. The top row shows the spherical projection error for different images sizes. As the input features are not relevant for this type of error, the value for $c_{in}$ is irrelevant. The bottom row shows the bird’s-eye projection error for using a single image plane and using multiple ones. It is important to note that the projection error highly depends on the used projection size. (**a**) $c_{in}\times 64\times 512$; (**b**) $c_{in}\times 64\times 2048$; (**c**) Single plane; (**d**) Multi plane.

Even though the results are very good in comparison to the benchmark, it must be noted that by decreasing the image size, the occlusion increases. Figure 5 shows how the occlusion affects the re-projection error. Since we want to fuse the approach and compensate for such errors, the results are acceptable for now. Nevertheless, we should be careful because post-processing becomes more difficult with an increasing number of projected errors. For example, using a NN method with k=8 neighbors could avoid the errors for the large image but not for the smaller one. It would need a larger amount of neighboring points, which would result in more computation during post-processing.

#### 5.4.2. Bird’s-Eye View

**Figure 6:**
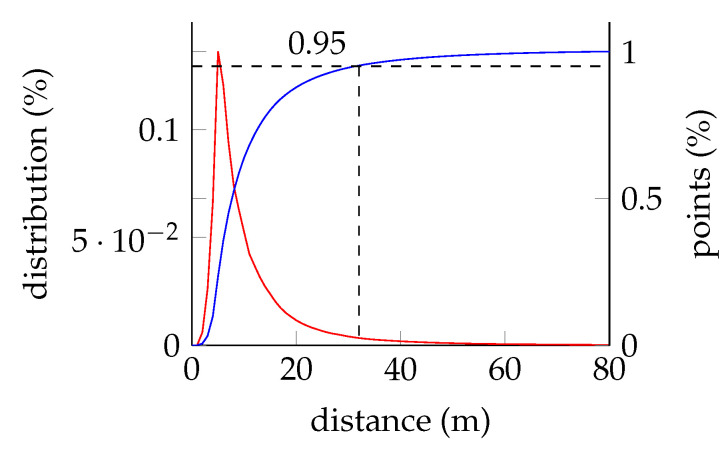
Depth distribution for the SemanticKITTI dataset. A total of 95% of all points are closer than 32 m.

To avoid too much unused space, we limited the range for points taking into account (−40,40) for *x* and *y* and (−3,3) for *z* as we normalize around the ground plane. In Figure 6, the depth distribution for all SemanticKITTI sequences up to sequence 10 are visualized. By taking only the points within the described range, we keep more than 97% of all points.

To compare the impact of using multiple projected images, we first trained our bird’s-eye model with three different grid sizes. The maximum height, the intensity, and the amount of points within each grid cell are used as input features. For the multi plane bird’s-eye view we voxelized the point cloud into 16 and 32 planes. As a feature, we simply projected the intensity value into each 3D cell. The results can be seen in Table 2. Using multiple planes highly increases the mIoU.

As expected, the occlusion error highly decreases by using multiple planes instead of single planes. Figure 5 shows that the terrain on the ground is labeled like the leaves of the tree. For the multiple plane approach, the ground gets labeled correctly, as it is not occluded. For the multi plane approach, occlusion only appears on top of the trunk.

#### 5.4.3. Cylindrical View

The feature map is created by the distance ρ, which has an approximated value range of (0,120). The value range of *z*, used for the multi plane bird’s-eye projection, is about (−3,3). Comparing the ranges with each other leads to the conclusion that we have to use a larger feature map. Taking the same size would end up in an unacceptable resolution of about 3.75 m. The experiment visualized in Figure 6 highlights that we can limit the values without losing much information. By reducing the range to (2,40) and doubling the input size for the feature map, the resolution decreases to 0.59 m or by doubling the feature map again to 0.3 m.

The experiments in Table 3 show significant improvement by using multiple planes as feature maps. However, the results are still not comparable to the other proposed views. The IoU values from Table 6 are clear that, for the most part, small objects, such as bicycles, are very poorly recognized.

### 5.5. Fused Projection

The baseline, as well as the NN fusion, are non-learnable approaches. For this reason, we will only show quantitative results within Section 5.6. For the approach of learning the fusion, we will take an input tensor of [3 × 64 × 512] or [3 × 64 × 2048] for the spherical branch and [32 × 400 × 400] or [32 × 512 × 512] for the bird’s-eye one.

#### 5.5.1. KPConv Fusion

First, we investigated how many layers are necessary. Since the bottleneck in this approach is in the calculation of the *k* NNs, we allow for up to three convolution layers. We remove the softmax function for each branch to directly use the output from the previously learned models. In Table 4, the results for different amounts of convolution layers and for different projection branches is shown. As already suspected, the increase of occluded points caused by the decreased image size causes problems for the fusion. Even though the fusion is able to compensate for most projection errors, the overall performance does not improve. By using a larger input image for both branches, the overall performance learned by the fusion highly increases.

#### 5.5.2. PointNet Fusion

**Figure 7:**
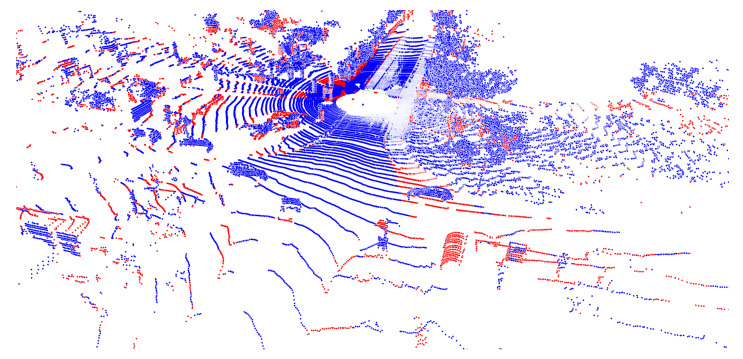
Map for disagreeing points. Agreement is visualized in blue and disagreement in red.

For the PointNet fusion, we first investigate how many points are left by taking only the disagreeing ones. Taking the smaller inputs ends up with an agreement rate of about 86.4% on the whole validation set. For the larger images, the agreement rate increases to 89.1%. As expected, the agreement rate is high for classes where both networks are strong. This leads to the fact that classes, such as road, car, or building, are no longer relevant within the disagreement map. An example of the map is shown in Figure 7.

As the PointNet model, we use only three layers of MLP’s, increasing the feature size up to 512 channels, to keep the fusion block small. Each layer is followed by batch normalization and ReLu as the activation function. The whole block has approximately 150 k parameters.

The results are shown in Table 5. By using the smaller input images, the performance is weak. Our PointNet model is not able to compensate errors, and does not provide any further advantage. The results are pretty much comparable to those with only just taking the agreeing points and assigning the disagreeing ones as unlabeled.

### 5.6. Comparison

The achieved results for each model on the SemanticKITTI validation split are shown in Table 6. The first part shows the results for our benchmark models. Within the second part, the performance of our single models is shown. The first 3D bird’s-eye uses a tensor of shape [32 × 400 × 400] and the second one [32 × 512 × 512]. Parts three and four are dedicated to the fusion results. The first fusion part uses the small and the second one the large images.

First, we compare the different views with each other. While the spherical one contains the highest information density, it suffers from deforming physical dimensions. The bird’s-eye and the cylindrical view, on the other hand, are keeping the dimensions but suffer from occluding points. Using multiple planes as input features addresses this problem but slightly increases the computation time. Nevertheless, the increase in performance is much more remarkable than the increase in computing time.

Fusing two views has proven to increase the overall performance. The baseline, the nearest neighbor, as well as the KPFusion, allow for error compensation. By using the larger input images and the KPConv as the fusion block, the highest overall performance can be achieved.

### 5.7. Generalization Analyses

**Figure 8:**
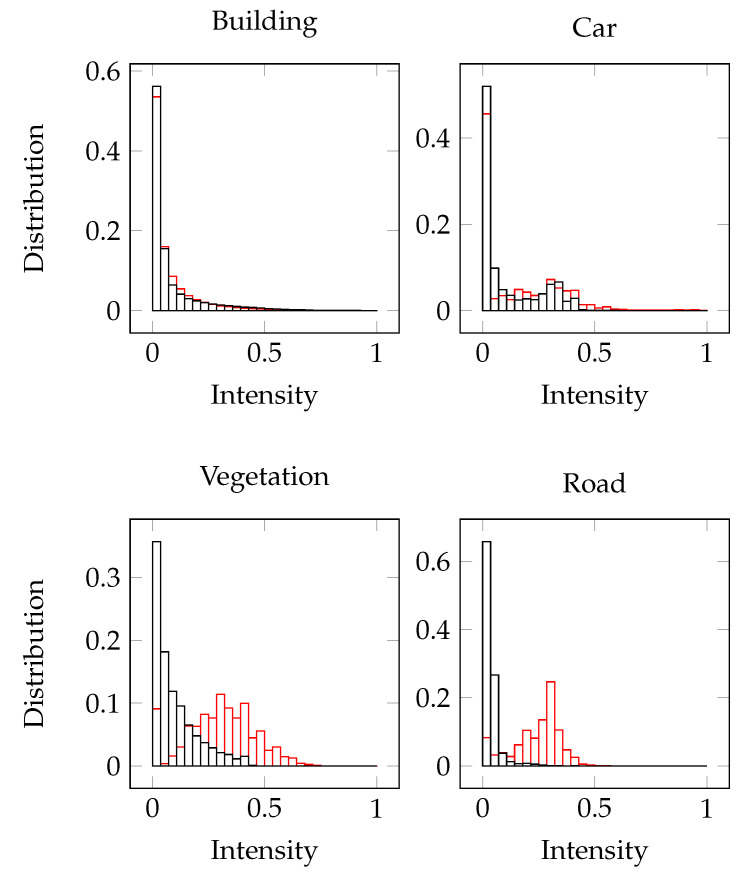
Intensity values for different classes and sensors (black for ParisLille, red for SemanticKITTI). The distribution for the classes building and car are comparable. Vegetation and road on the other hand highly differ.

We did figure out that the returned values for the intensity differ even in cases of using the same sensor brand. All Velodyne HDL64 are calibrated individually, making the transfer to a newer sensor almost impossible. To address this problem, the distribution of the normalized values ascribed to the intensity is visualized in the following Figure 8. The red histogram shows the intensity distribution for the SemanticKITTI and the black histogram for the ParisLille data. While the intensity values are comparable for the classes car and building, the values are different for the classes road and vegetation.

**Figure 9:**
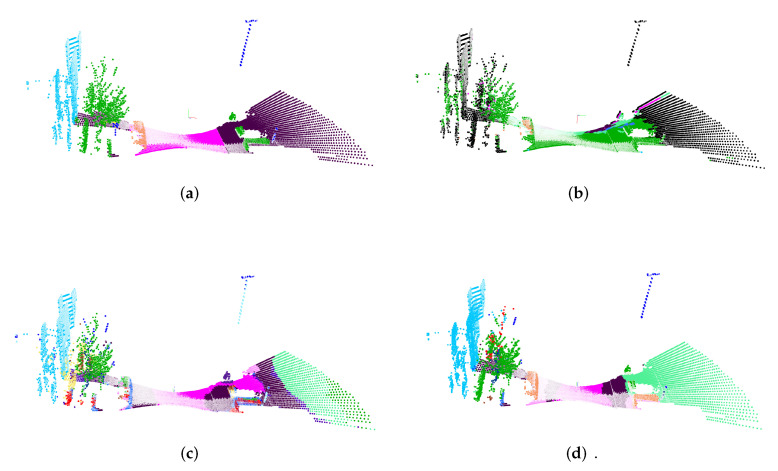
Results on ParisLille data. Using the best-performing single projection approach ends up in a very poor performance (**b**). The bird’s-eye projections are generally far better but are still worse than on the trained data. (**a**) Ground truth; (**b**) Prediction sphere; (**c**) Prediction bird’s-eye; (**d**) Prediction 3D bird’s-eye.

To still investigate the generalization to new setups, we have decided to remove the intensity channel and to use only the points itself, even if we could show that this channel has high information content. To evaluate the models, we use the sequence 01, 05, 13, and 23. Some classes are not included within this data set. Therefore, these classes are ignored within the mIoU. The results for the experiments are shown in Table 7. Column SK indicates the results that were achieved on the validation set from the SemantiKITTI data. Due to the different sensor setup, the bird’s-eye projection without the plane detection achieves poor results. Adding this feature increases the mIoU, but the performance is still poor compared to the results achieved within the SemanticKITTI evaluation. Even the multi plane bird’s-eye projection is not able to further improve the performance. Nevertheless, the model with the highest score on the SemanticKITTI evaluation has the poorest performance. An example for the predictions is given in Figure 9.

Carla offers high flexibility, but so far, there is no intensity value available for the simulated LiDAR module. Further, the output point cloud and the projections highly differ compared to a real sensor. Directly applying projection-based approaches has proven to be difficult. In [55], CycleGANs [56] are used to learn a sensor model for generating realistic bird’s-eye view images from the simulated LiDAR.

The experiment illustrates the biggest disadvantage of the projection-based approach. They highly depend on the used sensor and are not able to circumvent the problem of being invariant to permutation.

## 6. Conclusions

Within this work, we have investigated different projection-based 3D semantic segmentation approaches. First, we have compared different projections with each other and clarified how each one can achieve reasonable results. Even though the spherical projection is the most suitable for the given setup, we were able to make the bird’s-eye competitive by keeping it more flexible. The biggest advantage of this method is being fast and accurate at the same time. The biggest disadvantage, on the other hand, is the dependency on the sensor and the point of leaving out geometrical information. Afterward, we have shown that it is possible to fuse multiple projections and to profit from different view angles. Furthermore, we have shown that there are different ways to perform the fusion, while there are certainly other approaches that we have not considered in this work. Nevertheless, the fusion comes with a cost. The main advantage of the projection-based methods of being fast no longer applies. One of the biggest bottlenecks is the dependency on knowing the nearest neighbors. This leads to the conclusion that the approach can not be applied for real-time segmentation for now but is suitable for precise offline tasks.

## Figures and Tables

**Table 1:** Comparison of different input channels for the SalsaNext architecture. All results are without any post-processing steps. The projection of the coordinates x,y, and *z* does not bring any advantages, but the use of the surface normals *n* slightly increases the performance.

Input [$c_{\mathrm{in}}\times 64\times 512$]	$c_{\mathrm{in}}=d$	$c_{\mathrm{in}}=d,i$	$c_{\mathrm{in}}=d,i,n$	$c_{\mathrm{in}}=d,i,x,y,z$
mIoU val (%)	50.07	52.65	53.6	51.4

**Table 2:** Bird’s-eye projection results. Single plane projections use the maximum height, the corresponding intensity value, as well as the amount of points within each cell.

Planes	Single Plane	Multiple Planes	
Input	[3 × 256 × 256]	[16 × 256 × 256]	[32 × 256 × 256]
ine mIoU (%)	27.1	38.1	40.7
Inference (ms)	15	19	25
ine Input	[3 × 400 × 400]	[16 × 400 × 400]	[32 × 400 × 400]
mIoU (%)	31.1	43.6	48.1
Inference (ms)	22	23	49
ine Input	[3 × 512 × 512]		[32 × 512 × 512]
mIoU (%)	34.3		50.2
Inference (ms)	33		114

**Table 3:** Cylindric projection results. Even if the idea of using multiple planes brings a high improvement in performance, it is not comparable with the other proposed projections.

Planes	Single Plane	Multiple Planes	
Input	[3 × 64 × 512]	[64 × 64 × 512]	[128 × 64 × 512]
ine mIoU (%)	17.9	31.3	37.1
inference (ms)	11	21	47

**Table 4:** Parameter and performance for KPConv-based fusion. The fusion struggles to handle large projection errors. Using larger projections to decrease the projection error brings significant improvement for the fusion (Bird’s-Eye: 50.1, Spherical: 54.8).

	[32 × 400 × 400]		
	[3 × 64 × 512]		
Architecture	1 layer	2 layers	3 layers
	[15, 40, 256]	[15, 40, 64]	[15, 40, 64]
		[15, 64, 128]	[15, 64, 128]
			[15, 128, 256]
ine Parameter	153,600	161,280	652,800
ine mIoU (%)	51.5	52.9	53.4
inference (ms)	312	331	386
ine	[32 × 512 × 512]		
	[3 × 64 × 2048]		
ine mIoU (%)			60.7
inference (ms)			445

**Table 5:** Performance for PointNet-based fusion. Using larger projections to reduce projection error improves the performance of the fusion but is still worse than using single projections.

Input	[32×400×400]	[32×512×512]
	[3×64×512]	[3×64×2048]
mIoU (%)	43.4	50.1
inference (ms)	291	342

**Table 6:** Results of SemanticKITTI validation split. Section 1 shows relevant state-of-the-art methods and their results. Section 2 shows our single projections. The fusion approaches are shown in Section 3 and Section 4. Note that the values differ from the published values. This is because the validation data has been used, and there is no post-processing. For the fusion approach, the NN calculation is included within the inference time, and the architectures are not optimized.

Method	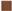 Car	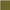 Bicycle	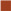 Motorcycle	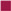 Truck	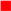 Other Vehicle	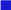 Person	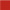 Bicyclist	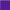 Motorcyclist	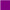 Road	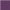 Parking	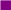 Sidewalk	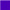 Other Ground	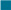 Building	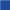 Fence	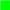 Vegetation	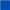 Trunk	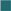 Terrain	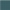 Pole	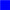 Traffic Sign	mIoU	Mean Time
RangeNet21	0.84	0.24	0.34	0.3	0.2	0.35	0.45	0.0	0.93	0.43	**0.8**	0.01	0.79	0.47	0.81	0.48	0.71	0.39	0.35	0.47	76
SalsaNext	0.86	0.39	0.42	0.78	0.42	0.62	0.68	0.0	**0.94**	0.42	**0.8**	0.04	0.80	0.48	0.80	0.58	0.64	0.47	0.44	0.55	52
PolarNet (cart)	0.93	0.27	0.50	0.47	0.27	0.53	0.72	0.0	0.89	0.36	0.72	**0.95**	0.89	0.46	0.86	0.6	0.74	0.53	0.43	0.53	39
Bird’s-eye	0.8	0.04	0.11	0.06	0.12	0.17	0.36	0.0	0.83	0.22	0.62	0.92	0.85	0.17	0.62	0.37	0.65	0.22	0.19	0.34	33
3D bird’s-eye	0.92	0.11	0.25	0.64	0.3	0.32	0.69	0.0	0.88	0.34	0.69	0.0	0.86	0.35	0.82	0.52	0.7	0.47	0.26	0.48	49
3D bird’s-eye	0.9	0.2	**0.68**	0.53	0.18	0.45	0.72	0.0	0.89	0.41	0.71	0.0	0.89	0.47	0.86	0.58	0.74	0.51	0.4	0.5	114
3D cylindrical	0.84	0.1	0.1	0.1	0.26	0.25	0.25	0.0	0.84	0.17	0.63	0.0	0.77	0.32	0.79	0.45	0.66	0.39	0.23	0.37	47
SalsaNext [3 × 64 × 512]	0.89	0.34	0.52	0.76	**0.46**	0.47	0.5	0.0	0.93	**0.45**	0.78	0.0	0.77	0.5	0.8	0.5	0.67	0.34	0.4	0.53	**11**
Baseline	0.91	0.32	0.45	**0.81**	0.38	0.42	0.64	0.0	0.91	0.41	0.75	0.0	0.88	0.51	0.85	0.55	0.71	0.46	0.34	0.54	240
KPFusion	0.93	0.35	0.26	0.62	0.4	0.38	0.65	0.0	0.89	0.37	0.72	0.0	0.88	0.51	0.86	0.59	0.72	0.55	0.46	0.53	386
PointNetFusion	0.91	0.06	0.17	0.66	0.36	0.2	0.09	0.0	0.89	0.34	0.71	0.0	0.86	0.41	0.83	0.36	0.7	0.32	0.35	0.43	291
NN k=8	0.92	0.32	0.47	0.8	0.4	0.44	0.66	0.0	0.91	0.42	0.75	0.0	0.88	0.5	0.85	0.56	0.71	0.49	0.35	0.55	327
KPFusion	**0.94**	**0.4**	0.42	0.73	0.42	**0.67**	**0.8**	0.0	**0.94**	0.43	**0.8**	0.0	**0.9**	**0.59**	**0.89**	**0.7**	**0.78**	**0.59**	**0.49**	**0.61**	445
PointNetFusion	0.9	0.19	0.08	0.3	0.22	0.51	0.7	0.0	0.91	0.36	0.76	0.2	0.88	0.5	0.87	0.61	0.76	0.57	0.45	0.5	342

**Table 7:** Evaluation of models on new sensor and sensor setup. This table points out the dependency on the used setup. Within column SK, the validation results for the SemanticKITTI dataset are shown.

		01	05	13	23	SK
Bird’s-eye (no RANSAC)	mIoU	8.9	8.7	7.9	6.5	-
	Acc	35	30.3	34.3	21.5	-
Bird’s-eye	mIoU	17.8	15	14.8	16.2	32.3
	Acc	50	54.6	53.1	45.4	70.6
3D bird’s-eye	mIoU	19.4	18.7	12.9	12.5	40.3
	Acc	66.9	69.3	55.5	51.3	81.3
Spherical	mIoU	6.3	4.8	5.8	5	50.1
	Acc	12.4	9.2	12.6	16.2	88.2

## Data Availability

The data used are referenced in the article.

## Abbreviations

The following abbreviations are used in this manuscript:

LiDAR	Light Detection and Ranging
kNN	k Nearest Neighbor
NN	Nearest Neighbor
MLP	Multilayer perceptron
RANSAC	RANdom SAmple Consensus
K	Kernel
D	Dilation
ReLu	Rectified Linear Unit
SGD	Stochastic gradient descent
IoU	Intersection over Union
mIoU	Mean Intersection over Union
Acc	Accuracy
